# Physiological and molecular implications of plant polyamine metabolism during biotic interactions

**DOI:** 10.3389/fpls.2014.00095

**Published:** 2014-03-18

**Authors:** Juan F. Jiménez-Bremont, María Marina, María de la Luz Guerrero-González, Franco R. Rossi, Diana Sánchez-Rangel, Margarita Rodríguez-Kessler, Oscar A. Ruiz, Andrés Gárriz

**Affiliations:** ^1^División de Biología Molecular, Instituto Potosino de Investigación Científica y Tecnológica, San Luis PotosíMéxico; ^2^UB3, Instituto de Investigaciones Biotecnológicas, Instituto Tecnológico de Chascomús, Universidad Nacional de San Martín, Consejo Nacional de Investigaciones Científicas y TécnicasChascomús, Argentina; ^3^Facultad de Ciencias, Universidad Autónoma de San Luis PotosíSan Luis Potosí, México; ^4^UB1, Instituto de Investigaciones Biotecnológicas, Instituto Tecnológico de Chascomús, Universidad Nacional de San Martín, Consejo Nacional de Investigaciones Científicas y TécnicasChascomús, Argentina

**Keywords:** polyamines, spermidine, spermine, putrescine, thermospermine, plant pathogen, mutualism

## Abstract

During ontogeny, plants interact with a wide variety of microorganisms. The association with mutualistic microbes results in benefits for the plant. By contrast, pathogens may cause a remarkable impairment of plant growth and development. Both types of plant–microbe interactions provoke notable changes in the polyamine (PA) metabolism of the host and/or the microbe, being each interaction a complex and dynamic process. It has been well documented that the levels of free and conjugated PAs undergo profound changes in plant tissues during the interaction with microorganisms. In general, this is correlated with a precise and coordinated regulation of PA biosynthetic and catabolic enzymes. Interestingly, some evidence suggests that the relative importance of these metabolic pathways may depend on the nature of the microorganism, a concept that stems from the fact that these amines mediate the activation of plant defense mechanisms. This effect is mediated mostly through PA oxidation, even though part of the response is activated by non-oxidized PAs. In the last years, a great deal of effort has been devoted to profile plant gene expression following microorganism recognition. In addition, the phenotypes of transgenic and mutant plants in PA metabolism genes have been assessed. In this review, we integrate the current knowledge on this field and analyze the possible roles of these amines during the interaction of plants with microbes.

## INTRODUCTION

The aliphatic polycationic compounds collectively known as polyamines (PAs) have proven to be essential for growth and development in all living organisms (Cohen, 1998). PAs participate in many fundamental processes such as chromatin condensation, DNA replication and transcription, RNA processing, protein synthesis and post-translational modification (Igarashi and Kashiwagi, 2000; Childs et al., 2003; Wallace et al., 2003). The most common PAs in nature are the diamine putrescine (Put), and those traditionally called higher PAs as the triamine spermidine (Spd) and the tetraamines spermine (Spm) and thermospermine (TSpm). In addition, other compounds are also regarded as PAs, such as the diamines 1,3-diaminopropane (DAP) and cadaverine (Cad). Uncommon PAs show a limited distribution in nature and consists of Spd and Spm-related molecules as nor-Spd, nor-Spm, homo-Spd, homo-Spm, and longer penta- and hexaamines (Bagni and Tassoni, 2001; Fuell et al., 2010; Sagor et al., 2013).

In plant tissues, the relative abundance of PAs depends on the species and the developmental stage. They are detected in actively growing plant tissues and in plants exposed to biotic or abiotic stress (Jimenez-Bremont et al., 2007; Rodriguez-Kessler et al., 2008; Alcázar et al., 2010; Gill and Tuteja, 2010; Hussain et al., 2011). It has been demonstrated that PAs are important for the normal course of diverse plant ontogenic processes such as cell division and elongation, organogenesis and somatic and zygotic embryogenesis (Hummel et al., 2004; Paschalidis and Roubelakis-Angelakis, 2005; Mattoo et al., 2010). In general terms, the levels of Put in plants are higher than those of the other PAs (Cohen, 1998). Cad can also be found in considerable high concentrations even though it seems to be limited to legumes and a few species belonging to other plant families (Tomar et al., 2013). Additionally, PAs may exist in soluble as well as insoluble fractions. The soluble fraction consists of free PAs and PAs conjugated to organic acids, mainly hydroxycinnamic acid. In turn, the insoluble fraction is formed by PAs bound to macromolecules such as proteins, cell wall polysaccharides and plasma membrane (Martin-Tanguy, 2001).

Although the exact mechanism of action of PAs remains still elusive, many authors have proposed that the spatial separation of their positive charges under physiological pH plays a crucial role. This feature gives them the capacity to bind negatively charged molecules such as nucleic acids, phospholipids, and proteins; and it is thought that by these means PAs affect the structure and function of these macromolecules. Moreover, the formation of these compounds protects macromolecules from degradation and modification (Ruiz-Herrera et al., 1995; D’Agostino et al., 2005).

There is a considerable amount of information about the role played by PAs during the interactions of plants with either pathogenic or beneficial microbes. These data indicate that changes in PA metabolism constitute a key adaptive response of the plant, and that their occurrence determines the development of the interaction. In turn, evidence suggests that some microorganisms are able to perturb plant PA metabolism in order to adjust it to their own requirements.

In this review, we integrate the current knowledge on the modulation of plant PA metabolism upon pathogen recognition and its importance for plant defense, giving particular emphasis in the advances achieved by the scientific community in the last few years. In addition, we discuss the importance of PAs in beneficial plant interactions, as those established by nitrogen-fixing bacteria and mycorrhizal fungi. The impact of biotechnological approaches and genetic engineered plants with altered PA levels in plant–microbe interactions is also reviewed.

## PA METABOLISM IN PLANTS

The concentration of PAs is finely controlled in eukaryotic cells in order to fulfill the requirements at any time. Precise regulation of PA levels is important since not only its depletion, but also extensive PA accumulation may be deleterious (Ditomaso et al., 1989; He et al., 1993; Poulin et al., 1993). Therefore, several processes such as PA synthesis, catabolism, transport, compartmentalization, and conjugation act in concert to maintain the concentration of these compounds in suitable levels (reviewed by Kusano et al., 2007; Carbonell and Blázquez, 2009). We briefly summarize below the principal aspects of plant PA metabolism.

## BIOSYNTHESIS OF PAs

In plants, the synthesis of Put may be conducted by two pathways (Martin-Tanguy, 2001). One of these metabolic routes starts with the decarboxylation of the amino acid arginine by the enzyme arginine decarboxylase (ADC, EC 4.1.1.19), located mainly in the chloroplast in photosynthetic cells but directed to the nucleus in non-photosynthetic cells (Borrell et al., 1995; Bortolotti et al., 2004). This reaction renders agmatine, which is then metabolized to N-carbamoylputrescine by agmatine iminohydrolase (AIH, EC 3.5.3.12). Consequently, N-carbamoylputrescine is converted in Put by the enzyme N-carbamoylputrescine amidohydrolase (NCPAH, EC 3.5.1.53). The other metabolic pathway begins with the amino acid ornithine converted into Put in a single-step reaction catalyzed by the enzyme ornithine decarboxylase (ODC, EC 4.1.1.17), which is mainly found in the plant cytosol. The synthesis of higher PAs requires decarboxylated S-adenosylmethionine (dcSAM) as aminopropyl donor, generated by the action of the enzyme S-adenosylmethionine decarboxylase (SAMDC, EC 4.1.1.50). dcSAM is then used by the enzyme spermidine synthase (SPDS, EC 2.5.1.16) to add an aminopropyl moiety to Put, hence forming Spd. In a consecutive aminopropylation reaction, the enzyme spermine synthase (SPMS, EC 2.5.1.22) adds an aminopropyl group to Spd converting it to Spm. Alternatively, thermospermine synthase (tSPMS, EC 2.5.1.79) forms TSpm by adding the aminopropyl group to the opposite end of the molecule of Spd.

The relative importance of ADC and ODC activities for Put synthesis varies according to the plant species and the tissue involved (Cohen, 1998; Hanfrey et al., 2001; Flemetakis et al., 2004). In some plant species the *ODC* gene is absent, as occur in many members of the *Brassicaseae* family including *Arabidopsis thaliana*, and in non-vascular plants such as *Physcomitrella patens* (Jiménez-Bremont et al., 2004; Fuell et al., 2010). In addition, whereas in most plants there is only one gene coding for ADC, two paralogous genes with different patterns of expression occurs in *Brassicaseae* members (Galloway et al., 1998; Hummel et al., 2001). The disruption of each *ADC* gene in *Arabidopsis* does not cause a major phenotypic change, but knocking down both has a lethal effect (Urano et al., 2005).

The *Arabidopsis* genome contains four genes coding for SAMDCs, two SPDSs, one SPMS and one tSPMS (known as *ACAULIS5*; Fuell et al., 2010). SPDS and SPMS seem to be localized in the cytoplasm and the nucleus (Belda-Palazón et al., 2012), while SAMDC appears to be mostly cytoplasmic (Torrigiani et al., 1986; Belda-Palazón et al., 2012). Plant SAMDCs are usually regulated at the translational level by PAs (Hanfrey et al., 2002, 2003). It has been shown that a small upstream open reading frame (uORF) in the SAMDC mRNA 5′ leader sequence is responsible for RNA translation repression mediated by PAs. This mechanism regulates the availability of dcSAM for Spd and Spm synthesis, which seems to constitute the main point of regulation for the synthesis of higher PAs in these organisms (Fuell et al., 2010). The double mutants *atsamdc2*(*bud2*)-*atsamdc1* (Ge et al., 2006) and *atspds1-atspds2* (Imai et al., 2004b) are embryo lethal, indicating that higher PAs are essential for plant embryogenesis. This effect could be due to a deficiency in Spd synthesis and not Spm, since no phenotypic effect is evident in *spms* plants (Imai et al., 2004a). In turn, disruption of the *ACAULIS5* gene in *Arabidopsis* plants lead to a notable dwarf phenotype, demonstrating the importance of TSpm synthesis for plant growth (Imai et al., 2004a).

## CATABOLISM OF PAs

Polyamine catabolism is mediated by diamine oxidases (DAOs, EC 1.4.3.6) and PA oxidases (PAOs, EC 1.5.3.3). DAOs are homodimers with copper as the prosthetic group. These enzymes show a preference for diamines such as Put and Cad, and have low affinity for Spd and Spm. The oxidation of Put renders H_2_O_2_, NH_4_^+^ and γ -aminobutanal, the latter being spontaneously converted to Δ ^1^-pyrroline. DAOs from legumes and *Arabidopsis* have been described to be directed to the extracellular space, remaining in this compartment loosely associated to cell walls (Federico and Angelini, 1991; Moschou et al., 2008b). Recently, it was demonstrated that *Arabidopsis* has at least ten putative *DAO* genes, four of which have been characterized: *AtCuAO1* and *AtAO1* encode for apoplastic proteins, whereas the products of *AtCuAO2* and *AtCuAO3* are found at the peroxisome (Planas-Portell et al., 2013). Interestingly, *DAO* genes show different expression profiles in response to external stimuli (Planas-Portell et al., 2013).

Polyamine oxidases use FAD as cofactor and oxidize mainly Spd and Spm. These enzymes may be involved in two different catabolic pathways: (1) terminal catabolism and (2) back-conversion of PAs. PAOs acting in the terminal catabolism pathway oxidize Spd or Spm using molecular O_2_ as electron donor. In this case, DAP and H_2_O_2_ are released as final products, besides either 4-aminobutanal or (3-aminopropyl)-4-aminobutanal depending on whether Spd or Spm act as substrate, respectively. Alternatively, enzymes back-converting PAs transform Spm into Spd, and Spd into Put with the concomitant production of 3-aminopropanal and H_2_O_2_. Both types of reactions produce H_2_O_2_ as final product, which plays an essential role in plant–microbe interactions. Five genes coding for PAOs have been identified in *Arabidopsis*, showing cytosolic (AtPAO1 and AtPAO5) and peroxisomal (AtPAO2, AtPAO3, and AtPAO4) localization (Moschou et al., 2008c; Takahashi et al., 2010; Fincato et al., 2011). It has been demonstrated that AtPAO1 to AtPAO4 catalyze the back conversion of PAs (Tavladoraki et al., 2006; Kamada-Nobusada et al., 2008; Moschou et al., 2008c; Fincato et al., 2011). The peroxisomal back-converting pathway has also been described in rice (Ono et al., 2012), even though all other enzymes characterized in monocotyledonous plants so far are involved in the terminal catabolic pathway (Tavladoraki et al., 1998; Šebela et al., 2001). It was recently found that *AtPAO2* in *Arabidopsis* is negatively modulated in a PA-dependent manner by a uORF located in the 5′-UTR sequence (Guerrero-González et al., 2014). A comparative analysis of several PAO transcripts from different plant species indicated that uORF sequences are very conserved, suggesting a common regulatory mechanism for these enzymes.

## TRANSPORT AND CONJUGATION OF PAs

The transport of PAs into different cell compartments is a crucial step to regulate several cell processes (Igarashi and Kashiwagi, 2010; Mulangi et al., 2012b). Only PA importers have been characterized so far at the functional level in plant cells. The first evidence of PA import was found in carrot protoplasts and isolated vacuoles, and it was suggested that PA entrance is driven by the transmembrane electrical gradient and a possible antiport mechanism (Pistocchi et al., 1988). It was found that external pH and the concentration of calcium modulate PA uptake (Antognoni et al., 1994; Pistocchi et al., 1988). Recently, the identification and characterization of specific importers has been achieved in rice and *Arabidopsis* (Fujita et al., 2012; Mulangi et al., 2012a). The first of these proteins reported was OsPUT1 (polyamine uptake transporter) from rice. Radiological uptake and competitive inhibition experiments in a heterologous system revealed that OsPUT1 functions mainly as a Spd importer and that the gene is expressed in all tissues except for mature roots and seeds (Mulangi et al., 2012b). Five additional importers from *Arabidopsis* and rice were described to form part of the same clade with *OsPUT1* and show high affinity for Spd (Mulangi et al., 2012a). A LAT (L-type amino acid transporter) protein in *Arabidopsis* named RMV1 is involved in the proton-dependent incorporation of PAs with a high affinity for Spm (Fujita et al., 2012). Lastly, PA transport into phloem and xylem tissues has been reported (Friedman et al., 1986), but the cell components involved in this processes have not yet been characterized.

Conjugation of PAs is an additional mechanism used by plant cells to regulate the intracellular concentration of the free forms, and these conjugates may comprehend up to 90% of the total PA cell content in some plant species (Bagni and Tassoni, 2001). It has been shown in *Nicotiana attenuata* that different hydroxycinnamoyl transferases (HCTs, EC 2.3.1.138) catalyze the conjugation of caffeoyl, cinammoyl, and feruloyl-CoA to Put or Spd (Onkokesung et al., 2012). It is unknown whether the deconjugation of these compounds is catalyzed by the same transferases. Interestingly, the genes coding for HCTs in *N. attenuata *are induced by the transcription factor R2R3-NaMYB8, which is known to mediate the defense responses against herbivore attack (Kaur et al., 2010).

## PAs AND PLANT–MICROBE INTERACTIONS

Plant PA metabolism undergoes remarkably changes during plant–microbe interactions (Walters, 2003; Hussain et al., 2011). The roles played by PAs during these processes are considerably intricate. This is due to the fact that PAs are not only essential to maintain cell viability, but function as signaling molecules regulating many of the responses that help the plant to cope with biotic stress. As will be discussed later, this effect may be exerted either directly by PAs or being mediated through their catabolism. The modulation of PA metabolism is so important for the outcome of the pathogenic interactions that not only the plant modifies PA concentration in response to pathogens, but some microorganisms have developed mechanisms to induce modifications of PA levels in host tissues. Therefore, it seems that when plants are attacked by pathogens, the organism that takes control of the PA machinery has a great opportunity to take the lead. The interaction of plants with beneficial microorganisms also induces changes in PA metabolism, and the establishment of mutualism with symbionts such as Rhizobia and Mycorrhizae appear to depend on PA levels. In the following section we aboard the evidences generated so far regarding the role played by PAs in plant associations with both pathogenic and beneficial microorganisms.

## CHANGES IN PLANT PA METABOLISM DURING PATHOGENIC INTERACTIONS

Research indicates that PAs participate actively during the interactions between plants and pathogens. It has been shown that PAs levels and the activity of PA metabolic enzymes augment in infected tissues during microbial colonization, which seems to be independent of the nature of the pathogen, i.e., biotroph or necrotroph (Walters, 2003; Hussain et al., 2011).

Biotrophic microorganisms feed on nutrients produced by the host living cells (Glazebrook, 2005) and the establishment of this type of interaction leads to the accumulation of PAs in plants. For instance, Greenland and Lewis (1984) reported an increase of Spd in barley (*Hordeum vulgare *L.) leaves infected with the rust fungus *Puccinia hordei*, while Spd and Put were increased in this plant species during the infection caused by the powdery mildew fungus *Blumeria graminis *f. sp. *hordei *(Walters et al., 1985). Similar effects were demonstrated to occur in the interaction between wheat and *Puccinia graminis* f. sp. *tritici* (Machatschke et al., 1990; Foster and Walters, 1992) and in bean plants treated with fungal elicitors (Broetto et al., 2005). Importantly, the main increments in PA concentration during the interaction with biotrophs are found in leaf areas close to the pathogen entry point. These areas have been termed “green islands” and are correlated with the induction of photosynthesis and delay in senescence. The accumulation of PAs was explained on the basis of higher activities in plant PA biosynthetic enzymes, in particular that of ADC (Walters and Wylie, 1986; Coghlan and Walters, 1990). Nevertheless, this is not necessarily true for all the interactions involving microorganisms of this kind. For instance, the ODC seems to be responsible for the increment of PAs in wheat leaves infected by* Puccinia graminis* f. sp. *tritici* (Foster and Walters, 1992). In this particular case, the authors reported a reduction of ADC activity but a significant induction of ODC activity in the pustules, the sites were fungal spores brake through the plant epidermis. On these grounds, it was hypothesized that the ODC from the pathogen would be responsible for PA production at the infection site.

Variations in PA levels between resistant and susceptible cultivars in response to pathogens has been described. In this trend, the analysis of two genotypes of barley during the interaction with *B. graminis* f. sp. *hordei *revealed that the resistant cultivar accumulates higher Put and Spd levels than the susceptible one following pathogen infection (Cowley and Walters, 2002a,b; Asthir et al., 2004). Similarly, PAs are accumulated in resistant (but not susceptible) tobacco plants in response to tobacco mosaic virus (TMV; Marini et al., 2001). Accordingly, highly susceptible sugarcane buds infected with the smut fungus *Ustilago scitaminea* reduce free PA contents while increase their conjugation to phenolic acids (Legaz et al., 1998). The authors attributed the susceptibility of the plant to a reduction in the availability of free phenolic compounds with importance for defense rather than a diminution in free PA concentration. In contrast,* Arabidopsis *ecotypes Col-0 and Bur-0, which are susceptible and partially resistant to *Plasmodiophora brassicae* infection, respectively, did not show significant differences in PA contents when challenged with the pathogen. However, susceptible plants displayed a transient accumulation of the PA precursor agmatine and strong arginase activity (the enzyme catalyzing the transformation of arginine in ornithine), whereas partially resistant plants showed continuous agmatine production and weak arginase activity. Based on these observations, it was proposed that symptom development is associated to differences in arginine metabolism and agmatine production (Jubault et al., 2008).

Many studies have shown that any situation leading to free PA accumulation in plants is usually accompanied of a rise in the concentration of conjugated PAs and the induction of PA oxidase activity. This is important because both phenomena could account for the improvement in plant tolerance. Thus, conjugated PAs are accumulated in plant tissues in response to viral and fungal infections (Walters, 2003; Rodriguez-Kessler et al., 2008; Muroi et al., 2009), and it was shown that these compounds affect microbial growth and prevent the development of disease in plants (Martin-Tanguy et al., 1976; Mackintosh et al., 1997; Walters et al., 2001). Muroi et al. (2009) reported the accumulation of conjugates of agmatine and Put in leaves of *A. thaliana* infected with *Alternaria brassicicola*. The importance of these compounds in the defense against this pathogen was clearly demonstrated by the interruption of the *AtACT* gene, encoding an enzyme that conjugates PAs to hydroxycinnamic acid, which enhanced the plant susceptibility. In addition, it has been suggested that PA conjugates contribute to the formation of a phenolic barrier that makes cell walls more resistant to microbial enzymatic hydrolysis (Walters et al., 2001). In turn, PA oxidation could also have a positive effect on plant defense. DAO and PAO activities were induced accompanying the accumulation of free PAs in tobacco and barley plants resistant to TMV and *B. graminis* f. sp. *hordei*, respectively (Marini et al., 2001; Cowley and Walters, 2002a,b). Interestingly, plant resistance to pathogens in these cases depends on the activation of the hypersensitive response (HR), which is characterized by host cell death at the infection site. As one of the key steps triggering the HR is the production of H_2_O_2_, it is conceivable that the activity of PA catabolic enzymes contributes to elicit this response. In addition, H_2_O_2_ may promote plant resistance by HR-independent mechanisms, involving cell wall reinforcement, MAPK activation, and modulation of gene expression (Cona et al., 2006a; Nanda et al., 2010). In this regard, PA oxidation was also induced in barley against an incompatible strain of *B. graminis*, where resistance is not associated to HR induction but depends on the ability to avoid penetration of leaf epidermal cells (Cowley and Walters, 2002a). In the same line of evidences, DAO activity is induced in chickpea in response to the necrotrophic pathogen *Ascochyta rabiei*, and its inhibition by 2-bromoethylamine reduces plant resistance (Rea et al., 2002). Necrotrophic pathogens kill the host cells to feed on the remaining, and it has been demonstrated that the activation of HR ultimately favors host colonization (Govrin and Levine, 2000). Therefore, it might be expected that the resistance to *Ascochyta rabiei* mediated by PA oxidation would be due to a mechanism other than HR.

## DIRECT MODULATION OF HOST PA METABOLISM BY PHYTOPATHOGENIC MICROBES

Plant tumor-like structures provoked by pathogens show a remarkably induction of PA synthesis and increase in PA concentration. For instance, PAs accumulate in turnip roots infected by *Plasmodiophora brassicae Woron*., a microorganism that produce hypertrophy and hyperplasia of infected tissues in members of the plant family *Brassicaceae* (Walters and Shuttleton, 1985). Similar increments in free and conjugated PAs were described in maize tumors produced by the fungus *Ustilago maydis* (Rodriguez-Kessler et al., 2008), where ADC and SAMDC activities played a major role. These observations raise an interesting question that has remained elusive so far: are pathogens able to modulate plant PA metabolism in their own favor? Recent studies demonstrate that it might be the case for some interactions. Cytokinins produced by *Rhodococcus fascians* induce Put accumulation in *Arabidopsis* by activating *ADC* expression, which contributes to symptom development (Stes et al., 2011). Moreover, PA synthesis is induced in cabbage protoplasts infected by TYMV and it was demonstrated that these newly synthesized PAs are incorporated by viral particles (Balint and Cohen, 1985). It has not been proven yet if this is triggered by signals generated by this pathogen to assure survival in the host cells. In turn, perhaps one of the most direct evidences demonstrating the modulation of plant PA metabolism by a pathogen is the interaction between the protein 10A06 secreted by the nematode *Heterodera schachtii* and the product of the *SPDS2* gene of *Arabidopsis*. Interestingly, expression of 10A06 in *Arabidopsis* enhanced plant susceptibility, which was associated to the activation of *SPDS2* gene expression, the increment in Spd concentration and the activation of PAO activity (Hewezi et al., 2010). Surprisingly, over-expression of *SPDS2* was sufficient to affect plant defense. A further examination of these phenotypes demonstrated that *10A06 *or* SPDS2* expression increases the abundance of several transcripts coding for host antioxidant enzymes. Based on this, the authors hypothesized that the induction of PAO activity imposes oxidative stress to plant cells and that this is counteracted by the activation of the antioxidant machinery, which might create a less hostile environment for the pathogen. In this same line, it was demonstrated that the protein C2 of the beet severe curly top virus (BSCTV) interacts with SAMDC1 in *Arabidopsis*, which contributes to increment plant susceptibility (Zhang et al., 2011b). In this case, it was found that C2 interacts with a region of SAMDC1 containing a PEST sequence (enriched in the amino acids Pro, Glu, Ser, and Thr), thereby attenuating its degradation at the proteasome. In this context, the stabilization of SAMDC activity leads to a low SAM/dcSAM ratio, thus reducing host and viral DNA methylation. It is though that this hypomethylated environment facilitates viral replication. Whether direct interaction with host PA metabolism enzymes is a general virulence mechanism for pathogens has not been further evaluated. Interestingly, a recent work by Kim et al. (2013a) demonstrated that the interaction between PA metabolic enzymes and pathogen effectors could also be used by plants to trigger defense. Thus, the effector protein AvrBsT of *Xanthomonas campestris* pv. *vesicatoria* is recognized by the ADC1 protein at the cytoplasm of pepper cells, and this interaction enhances AvrBsT-mediated cell death and plant resistance.

## TRANSGENIC MODULATION OF PLANT PA LEVELS TO IMPROVE THE TOLERANCE TO PATHOGENS

Several genetically engineered plant lines with altered PA levels show increased tolerance to microorganism attack. This fact constitutes another line of evidence demonstrating the importance of PAs during plant–pathogen interactions. In relation to this, tobacco plants over-expressing the human *SAMDC* gene accumulate free and conjugated PAs and show higher tolerance to pathogens (Waie and Rajam, 2003). Similarly, transgenic eggplants over-expressing the oat *ADC* gene acquired resistance to wilt disease caused by the fungus *Fusarium oxysporum *(Prabhavathi and Rajam, 2007)*. *These plants**showed increased ADC activity and accumulation of PAs, particularly the conjugated forms of Put and Spm. As the DAO activity was also enhanced, it was suggested that the acquisition of resistance might be due to an integrated mechanism involving PA biosynthesis and degradation rather than a single event. In turn, over-expression of human *SAMDC* gene in tomato conferred resistance to the fungal pathogens *F. oxysporum* and *Alternaria solani* (Hazarika and Rajam, 2011), and the over-expression of an apple *SPDS* gene in sweet orange enhances plant tolerance to *Xanthomonas axonopodis* pv. *citri* (Fu et al., 2011; Fu and Liu, 2013). At last, the over-expression of the *SPMS* gene in *A. thaliana* resulted in higher Spm levels and enhanced resistance against *Pseudomonas viridiflava* (Gonzalez et al., 2011). Promoting PA oxidation might also favor plant defense. Regarding this, the over-expression of an extracellular PAO from maize in tobacco plants leads to an increment in the expression of genes related to systemic acquired resistance and cell wall-based defense (Moschou et al., 2009). These changes confer disease resistance against *Pseudomonas syringae pv. tabaci* and *Phytophthora parasitica*, but not against infections caused by the* cucumber mosaic virus*.

## PAs AND DEFENSE HORMONE METABOLISM

Polyamine metabolism is modulated by exogenous application of phytohormones known to be associated to plant defense. However, only a few works addressed the relationship between PAs and defense hormones in a plant-pathogen system. For instance, treatment of barley primary leaves with methyl-jasmonate (MeJA) induces the increment in free and conjugated Put and Spd, as well as in ODC, ADC, SAMDC, and DAO activities (Walters et al., 2002). Conjugated PA accumulation was extended to non-treated leaves and correlated with a reduction in powdery mildew infection in these tissues. It was also shown that application of MeJA entails PA accumulation in loquat fruits and reduction of the symptoms associated to anthracnose rot caused by *Colletotrichum acutatum* (Cao et al., 2014). Similarly, treatment of wheat with MeJA increases the concentrations of both free and conjugated PAs and induces ODC and PAO activities. The change in PA metabolism was correlated with the induction of pathogenesis related (PR) proteins such as peroxidases and chitinases and a reduction in the incidence of infections by *Puccinia recondita* (Haggag and Abd-El-Kareem, 2009). The over-accumulation of conjugated PAs and induction of PA oxidation mediated by jasmonates (Jas) was also reported to occur in *Hycoscyamus muticus *L. root cultures, tobacco leaf disks and chickpea plants (Biondi et al., 2000, 2003; Biondi and Scaramagli, 2001; Rea et al., 2002). The effect of MeJA on conjugated PA levels is exacerbated in tobacco transgenic lines expressing a *SAMDC* gene in antisense, which showed low SAMDC activity and a relative accumulation of Put (Torrigiani et al., 2005). Interestingly, treatment of Arabidopsis plants with MeJA increases the expression of *ADC2* while *ADC1* remains unaltered, suggesting the existence of different regulatory pathway for both genes (Perez-Amador et al., 2002). Importantly, *ADC2* is regulated by a stress-induced kinase cascade involving MPK3 and MPK6 and is important for defense against *P. syringae* pv. tomato DC3000 (Kim et al., 2013b). Two *ODC* genes are also induced in tobacco in response to MeJA, but its importance for plant defense has not been explored (Xu et al., 2004). The effect of MeJA on PA metabolism may be quite different in other plant species. Thus, it has been shown that MeJA caused a transient inhibition at the transcriptional levels of the expression of *ADC*, * SAMDC, *and* SPDS* in rice (Peremarti et al., 2010). These data could be explained by the existence of contrasting regulatory mechanisms operating over the metabolic enzymes in different plant species. In addition, it should also be considered that the expression of PA metabolism genes not only depends on the action of one hormone, but it is influenced by several signaling pathways. Thus, it has been demonstrated that the effect of MeJA on ODC and SAMDC activities function synergistically with that of auxins in tobacco leaf disks, but it is counteracted by cytokinins (Biondi et al., 2003). In *Arabidopsis*, MeJA induces the expression of *CuAO1*, *CuAO2,* and *CuAO3*, and it was shown that *CuAO1* and *CuAO3* also respond to abscisic acid (ABA; Wimalasekera et al., 2011b; Planas-Portell et al., 2013).

Salicylic acid (SA) plays a fundamental role in plant defense (Glazebrook, 2005). Unfortunately, the current information concerning the relationship between SA and PAs is scarce. It has been shown that SA is able to induce the accumulation of PAs by activating the expression of *ADC* and *ODC* in maize, tobacco, and tomato (Németh et al., 2002; Jang et al., 2009; Zhang et al., 2011a). Nevertheless, it has not been evaluated yet whether JA and SA act synergistic on the activities of these enzymes. In chickpea plants, however, the application of SA repressed the induction of PA oxidation mediated by JA (Rea et al., 2002). In turn, ABA has been recently demonstrated to induce DAO expression in *Arabidopsis* (Wimalasekera et al., 2011b). ABA is also able to induce Put oxidation at the apoplast of *Vicia faba *(An et al., 2008). This was demonstrated to be important for stomatal closure, a known mechanism contributing to prevent plant colonization by bacterial pathogens. Future research is necessary to understand the connections between SA and ABA metabolism with PAs during plant biotic stress.

The infection of tomato and purple passion (*Gynura aurantiaca*) plants with the citrus exocortis viroid (CEVd) induces ethylene production and represses PR expression. This is accompanied by a reduction in ODC activity, which leads to a decline in Put contents (Belles et al., 1991, 1993). Importantly, the reduction in Put concentration is absent with the use of ethylene biosynthesis inhibitors or compounds that prevent the action of the hormone, suggesting that ethylene exerts an inhibitory effect on ODC activity. Conversely, PAs may also interfere with ethylene metabolism. This was demonstrated in a tomato transgenic line expressing the yeast *SPDS *gene and showing relatively high levels of Spd (Nambeesan et al., 2012). This line exhibits high susceptibility to the attack of the fungus *Botrytis cinerea*, which seems to be due to an attenuated expression of ethylene biosynthetic and signaling genes.

## PAs AS MEDIATORS OF PLANT DEFENSE ACTIVATION

Increasing evidences demonstrate that PAs and their catabolic products (i.e., H_2_O_2_) can act as defense signaling molecules. This function has been mostly assigned to Spm, even though it has been shown that in some systems either Put, Spd, or TSpm participate in defense activation.

During TMV infection, Spm is accumulated in the apoplast of tobacco. This change induces the expression of acidic and basic PRs in a SA-independent pathway and is associated to resistance to virus infection (Yamakawa et al., 1998). Further studies demonstrated that Spm oxidation at the apoplast is crucial to elicit defense responses, and that this pathway involves the activation of key MAPK proteins as SIPK and WIPK (Takahashi et al., 2003; Moschou et al., 2008a). This signaling cascade also requires the opening of the mitochondrial pore and Ca^2^^+^ influx in this organelle, suggesting that a disruption of the electrochemical potential at this membrane participates in the process. Gene expression analysis in different plant species showed that Spm mediates the induction of several defense genes, such as members of the hairpin-induced 1 (HIN1) family (Takahashi et al., 2004a), proteinase inhibitors (Raju et al., 2009), the transcription repressor ZFT1 (Mitsuya et al., 2007), and HR-associated factors (Takahashi et al., 2004b). In tobacco, the activation of the Spm pathway ultimately leads to the induction of programmed cell death, thus promoting resistance to TMV. In relation to this, it has been shown that Spm is able to activate *NHL10* expression in *Arabidopsis*, another gene induced during HR (Zheng et al., 2005). The involvement of Spm in defense gene activation may be even broader in this species, where 90% of the genes regulated by this PA also respond during the HR elicited by an avirulent virus (Sagor et al., 2009). Most of these genes are important for cell redox homeostasis, protein metabolism and plant defense. Recent evidences demonstrated that the response triggered by Spm is not only restricted to HR-related genes. In this regard, Gonzalez et al. (2011) evaluated the transcriptome of an *Arabidopsis* line over-expressing the *SPMS* gene, which showed high tolerance to the virulent bacteria* P. viridiflava *by mechanisms that are independent of HR induction. This analysis revealed that Spm accumulation induces the expression of genes involved in pathogen perception and defense, including several regulatory proteins such as transcription factors and kinases. By the use of a PAO inhibitor the authors showed that the induction in gene expression may be mostly explained through Spm oxidation, even though part of the response if carried out by the native non-oxidized PA.

Put and Spd were also described to participate in plant defense in *Arabidopsis* and tobacco, respectively. In this trend, Put treatment reduces pathogen susceptibility of an *ADC2* mutant of *Arabidopsis*, suggesting that this diamine is involved in defense responses (Kim et al., 2013b). On the other hand, Spd accelerated the induction of HR and improves the tolerance against TMV of tobacco leaf disks (Lazzarato et al., 2009). In addition, tobacco plants treated with Spd showed higher levels of SA and expression of PRs, besides the induction of systemic acquired resistance. Constitutive expression of a *SPDS* gene in sweet orange also modifies the expression of a large group of genes, many of them with a potential function in plant defense (Fu and Liu, 2013). However, as Put and Spd function as precursors of Spm, it is not clear whether these PAs by their self are able to induce plant defense or rather it is carried out after its conversion to Spm.

In turn, TSpm has received particular attention in the last years. It has been shown that it is equally efficient at inducing defense gene expression as its isomer Spm (Sagor et al., 2012; Marina et al., 2013). In fact, exogenous TSpm is able to restrict CMV multiplication in *Arabidopsis* (Sagor et al., 2012) and improve the resistance of this species to *P. viridiflava* (Marina et al., 2013). In agreement with this, the over-expression of *ACAULIS5* in *Arabidopsis* enhances tolerance to bacterial attack while null mutant lines are more susceptible. Importantly, the use of PA oxidase inhibitors impairs resistance in the *ACAULIS5* transgenic lines, suggesting that TSpm oxidation is crucial to elicit plant resistance (Marina et al., 2013). Other longer uncommon PA isomers (caldopentamine, caldohexamine, and homocaldopentamine) have recently been described to induce defense gene expression in an even stronger way than that of Spm. These molecules are also oxidized in plant tissues but at lower rates than other PAs (Sagor et al., 2013). In addition, they are able to induce the expression of NADPH oxidase genes, the enzyme that produce superoxide anion ($O_{2}^{-}$) and thus contribute to ROS accumulation.

Finally, it has been suggested that PA action is mediated through the generation of nitric oxide (NO), a key mediator of plant defense. This is based on studies in *Arabidopsis* seedlings where it was shown that exogenous Spd and Spm increased NO production (Wimalasekera et al., 2011a). However, the interplay between PA and NO metabolism during pathogenic interactions in plants has not been evaluated to the present.

Polyamine oxidation plays an essential role during PA signal transduction. Importantly, the activities of these enzymes are increased upon pathogen contact, particularly in resistant cultivars, and transgenic plants constitutively expressing PA oxidase genes present higher tolerance to pathogen attack (Yoda et al., 2006; Moschou et al., 2008a, 2009). Conversely, the use of oxidase inhibitors or oxidase gene deletion strongly impairs plant resistance (Yoda et al., 2009; Choi and Hwang, 2012). Because PAO and DAO activities result in the production of H_2_O_2_, a process that contributes to elicit host cell death, it has been proven that the importance of PA oxidation depends on the life-style of the pathogen. For instance, oxidation of PAs was demonstrated to be beneficial for plant defense in pathosystems involving biotrophic or hemibiotrophic pathogens (Yoda et al., 2006, 2009; Moschou et al., 2009). By contrast, the induction of host cell death by PA oxidation facilitated pathogen spreading in plants interacting with necrotrophic microorganisms (Marina et al., 2008). The activation of defense responses mediated by these enzymes may not be attributed only to H_2_O_2_ production, since other reaction products may give rise to intermediaries with a substantial role during biotic stress. For instance, Δ ^1^-pirroline derived from Put and Spd oxidation is further metabolized to γ-aminobutyric acid (GABA), a compound largely associated to plant defense (Bouche and Fromm, 2004). Additionally, DAP could be also converted in β -alanine and uncommon PAs, all of them proposed to be involved in plant resistance (Cona et al., 2006b).

In particular, PA oxidation might have an important role when occurring at the apoplast. In this trend, Marina et al. (2008) demonstrated that oxidase activities are induced in apoplast of tobacco leaf disks challenged with very dissimilar pathogens such as *P. viridiflava *and* Sclerotinia sclerotiorum*. By the use of specific inhibitors of PA oxidation, they showed that this is important to avoid colonization by the bacteria, which presents a biotrophic lifestyle, but favors the disease caused by the necrotrophic fungus *S. sclerotiorum*. Likewise, the extracellular diamine oxidase AtCuAO1 is induced in *Arabidopsis* by stress-related stimuli as treatment with SA, MeJA, flagellin, and ABA (Planas-Portell et al., 2013). In tobacco, the oxidation of apoplastic PAs induces the HR during TMV infection and this is essential for defense against the bacterium *P. syringae *pv*. tabaci* and the oomycete *Phytophthora parasitica *var*. nicotianae *(Yoda et al., 2003; Moschou et al., 2009). In this trend, the expression of an extracellular PAO from maize in tobacco cells is sufficient to induce programmed cell death when PAs are exogenously added (Rea et al., 2004). Besides the induction of HR, it has been demonstrated that the oxidation of PAs at the apoplast is important to strengthen cell wall during pathogen attack (Cona et al., 2006a; Angelini et al., 2010).

## PLANT PA METABOLISM IN THE INTERACTION WITH BENEFICIAL MICROORGANISMS

Many soil-borne microorganisms have been well studied for their beneficial effects on plant growth and are classified into different categories, as nitrogen-fixing bacteria (rhizobia), mycorrhizal fungi, plant growth promoting rhizobacteria, mycoparasitic fungi, etc (Mendes et al., 2013). The current evidence demonstrating the influence of PA metabolism in the establishment and development of beneficial associations is relatively scarce in comparison with that obtained from studies on pathogenic associations. The research conducted in this area has been mainly focused in two groups: the nitrogen-fixing bacteria and mychorrizal fungi.

## PAs AND NODULATION IN LEGUMES

Symbiotic nitrogen fixation results from the interaction between legume plants and soil-borne bacteria called rhizobia. This interaction leads to the formation of a novel plant organ, the root nodule, in which rhizobia differentiate into specific nitrogen-fixing bacteroids able to reduce atmospheric nitrogen to ammonia (Oldroyd and Downie, 2008). Many studies demonstrate that nodule initiation and development is regulated by the availability of nitrogen. In addition, it has been shown that changes in PA concentration might affect the control of root nodule number and biomass (Vassileva and Ignatov, 1999; Terakado et al., 2006).

It is well known that root nodules accumulate PAs to levels that are 5 to 10 times higher than in non-symbiotic organs, even though the composition of PAs depends on the legume species studied (Fujihara et al., 1994; Efrose et al., 2008). It has been proposed that PA accumulation in mature nitrogen-fixing nodules could be a mechanism to enhance nodule tolerance to the osmotic stress caused by the establishment of the symbiotic interaction. Studies of symbiosis in *Lotus japonicus* indicate the occurrence of a coordinated spatial and temporal induction of PA biosynthesis genes throughout the nodule life, where the expression of *LjSPDS* and *LjSPMS* is particularly high in the earlier stages of nodule development (i.e., 10 days post-infection) and decline thereafter. Interestingly, the expression pattern of these genes did not correlate with Spd and Spm levels, which are incremented in later stages of nodulation (Efrose et al., 2008). This observation may reflect higher Spd and Spm catabolism rates in early stages, in which H_2_O_2_ may promote cross-linking of a plant matrix glycoprotein associated with the lumen of the infection threads, the initial structure that allows rhizobia colonization of root hairs (Wisniewski et al., 2000). DAO deficient plant lines are less efficient in cross-linking the matrix glycoprotein but they does not show a difference in nodule formation when compared to control plants, suggesting that Put oxidation plays a minor role in nodule initiation (Wisniewski and Brewin, 2000). These lines though, are less sensitive to the inhibition of nodule formation that Put exerts on wild type plants. These data indicates that products derived from Put oxidation may delay nodule development.

In the nitrogen-fixing interaction between *Galega orientalis *and *Rhizobium galegae, *the exogenous application of PAs (mainly Put) at concentrations ranging from 10 to 50 μM exerts stimulatory effects in the number and biomass of nodules. In addition, it stimulates nitrogen fixation and enhances the ability of the *Rhizobium* strain HAMBI 540 to attach to *Galega* roots (Vassileva and Ignatov, 1999). However, the exposition of this plant species to higher concentrations of PAs (100 μM) impairs nodule function and originates specific structural abnormalities, indicating that a fine balance in PA concentration must exist in the plant-rhizobia interphase to avoid deleterious effects on nodules and roots.

The relative concentration of each PA appears to be relevant to control nodulation in legumes. In this regard, it has been demonstrated that the soybean supernodulating mutant En6500 maintains high Put levels but low concentrations of Spd and Spm in leaves, roots and nodules in comparison to its parent plant Enrei (Terakado et al., 2006). Importantly, treatment of leaves with an inhibitor of Spd synthesis increases nodule formation in Enrei, whereas Spd and Spm applied to foliar tissues reduce nodulation in both mutant and parental lines. The supernodulating phenotype showed by the mutant line is probably caused by a deficiency in the metabolism of brassinosteroid, a hormone that probably induces Spd synthesis. In fact, exogenous application of the brassinosteriod brassinolide not only reduces root nodule number in this mutant, but also restores Spd levels (Terakado et al., 2006).

Uncommon PAs may also have important functions in nodule development. These PAs have been found in legume root nodules, and in many cases their synthesis and/or accumulation was restricted to these organs (Fujihara, 2009). For example, the tetraamines homo-Spd and the Spm analog canavalmine were identified exclusively in senescent nodules of the sword bean *Canavalia gladiata* (Fujihara et al., 1986). Unsaturated analogs of Spd, such as *N*-(3-aminopropyl)-1,4-diamino-2-butene and N-(4-aminobutyl)-1,4-diamino-2-butene, have been identified in root nodules induced by *Bradyrhizobium japonicum* (Fujihara and Yoneyama, 1999). It is still not clear if these PAs are produced by rhizobia and exported to the plant or whether they are actually produced by the plant cells. In *Medicago sativa *nodules, it was found that the high content of homo-Spd is provided by the bacteroid (López-Gómez et al., 2014).

Likewise, metabolites derived from PA catabolism appear to be important for nodulation. For instance, it has been proposed that GABA (which may be produced from 4-aminobutanal) has a dual role, acting as signaling molecule during plant-bacteria communication and also functioning as an amino acid precursor in bacterial metabolism (Sulieman, 2011). In this trend, feeding with GABA into the phloem sap of *Medicago truncatula* plants causes a short-term increase in nodule activity and a remarkably increment in the amino acid and organic acid content in nodules (Sulieman and Schulze, 2010).

## PAs AS INDUCTORS OF THE PLANT-MYCORRHIZAL ASSOCIATION

Plant root symbioses with fungi, referred as mycorrhiza, occur in different forms (Parniske, 2008). In this mutualistic association, the fungi are adapted to live using nutrients provided by the plant, usually nitrogen and carbohydrates. In return, the fungus augments water and minerals availability for the plant. The establishment of this interaction is accompanied by a complex exchange of signaling molecules between the symbiont and the plant, and it has been suggested that PAs may play an important role in this process (Niemi et al., 2002, 2006).

Exogenous application of PAs has a positive effect during the formation of mycorrhizal symbiosis. El Ghachtouli et al. (1995) observed that PAs significantly increased the frequency of mycorrhizal colonization in pea roots. Similarly, application of Put and Spm to trifoliate orange (*Poncirus trifoliata*) seedlings increased mycorrhizal colonization by *Glomus versiforme*, whereas Spd did not influence this parameter (Wu and Zou, 2009). This study also showed that PA treatment enhances leaf Mn^2^^+^ and root Ca^2^^+^ uptake. Therefore, it was concluded that PAs stimulate the colonization of roots by the fungus, hence helping the host to absorb minerals. Moreover, it has been reported that the exogenous application of PAs in combination with mycorrhiza colonization alter the morphology of plant roots (Wu et al., 2012). Thus, *Citrus tangerine* plants treated with PAs were better colonized by the fungus *Paraglomus occultum* and showed an increment in root length, diameter, surface area, and volume, as well as higher root branching. Similarly, treatment of *Citrus* *limonia *seedlings with either Put or Spd increases root length and augments the mitotic index of root tip cells (Yao et al., 2010). These data indicates that PAs are involved in root elongation of citrus plants. Likewise, the exogenous addition of Put, Spm, and Spm at 1 μM improves rooting frequency and provokes root elongation in Virginia pine (*Pinus virginiana*; Tang and Newton, 2005).

Some evidence demonstrates that mycorrhizae formation can result in changes in both free and conjugated PA levels in plant tissues. Thus, Kytöviita and Sarjala (1997) observed that PA concentration (mainly Put and Spd) augmented in root tips of old pine (*Pinus sylvestris*) inoculated with *Suillus variegatus* in comparison with non-mycorrhizal tips. Total free PA pools were also increased in *Lotus glaber* following colonization by *Glomus intraradices*, augmenting the Spd/Spm to Put ratio. It has been suggested that this is one of the mechanisms by which mycorrhizal fungi enhance plant adaptation to saline soils (Sannazzaro et al., 2007). Up to now, little is known about the molecular events that precede the changes in the content of PAs during the establishment of mutualistic association with mycorrhiza-forming fungi.

## CONCLUDING REMARKS AND FUTURE PERSPECTIVES

**FIGURE 1:**
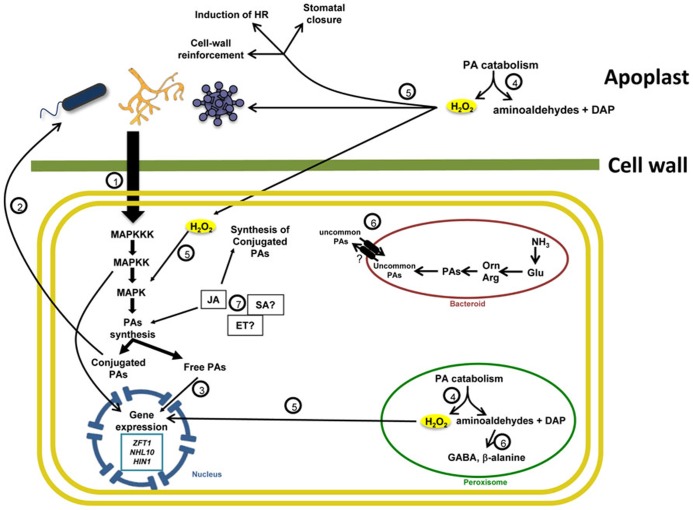
**Polyamine metabolism in plant-microorganism interactions.** (1) The presence of microorganisms is perceived by specific plant receptors that activate signal transduction pathways. Many MAP kinases, such as WIPK and SIPK, participate in these pathways stimulating defense gene expression and PA biosynthesis. This process leads to the accumulation of free and conjugated PAs. (2) Some conjugated PAs have an antimicrobial effect. (3) In turn, free PAs might contribute to the activation of gene expression directly or through their catabolism. (4) PA oxidation by DAO and PAO occurs in the peroxisome and apoplast, with the generation of H_2_O_2_, DAP and aminoaldehydes. (5) H_2_O_2_ acts as signaling molecule and stimulates defense gene expression. In addition, this molecule affect pathogen growth, induces cell-wall reinforcement and stomatal closure, and activates the HR. Other products from the oxidation of PAs may play a defensive role. For instance, aminoaldehydes are precursors for the biosynthesis of GABA in the cytosol and peroxisome, which participate in plant defense and plant-bacteria communication. DAP is a substrate for the synthesis of uncommon PAs and β-alanine, compounds that appear to have an important role during plant biotic stress. (6) Phytohormones such as JA contribute to defense responses by stimulating PA biosynthesis and conjugation. The role of SA and Ethylene in PA metabolism is less clear.

The experimental work conducted so far clearly indicates that PAs exert important biological activities during the establishment of plant–microbe interactions (see **Figure 1**). In brief, these data indicate that:

(a) Changes in PA levels occur in plants following microbe recognition. PA biosynthesis tends to be induced during this process, which usually lead to the accumulation of the free and conjugated forms of these compounds. The timeline of accumulation as well as the nature of the PAs accumulated depend on the type of the interaction being established and the species involved in the interaction
(b) The role of PAs during plant–microbe interactions might be the result of multiple mechanisms, i.e., their interaction with macromolecules, regulation of gene expression and protein stability, and the induction of defense signaling pathways.
(c) The accumulation of PAs is often accompanied by the induction of PA oxidation. PA catabolism is essential to trigger plant defense responses particularly when occurring at the apoplast, even though part of this response is also induced by non-oxidized PAs.
(d) The establishment of beneficial and pathogenic interactions might be dependent on PAs. Recent evidences suggest that both plant and microorganisms have evolved mechanisms to modulate PA metabolism in their own advantage.

In spite of the above mentioned, there are still several issues to be addressed in order to have a full understanding of the participation of plant PAs in the outcome of these associations. For instance, little is known about the mechanisms used by cells to transport different PAs to organelles and apoplast, and whether these compounds are actively transported between the plant cells and microbes. Additional research is needed to identify the molecular mechanisms that regulate PA metabolism and the main targets of PAs during the induction of plant defense, including the cross-regulation between PAs and phytohormones. These studies should involve not only the most common PAs referred in this review, but also other non-canonical molecules. At last, it is also essential to know the significance of the mechanisms deployed by microbes to perturb host PA metabolism, as well as the effects that PAs produced by these organisms may have on plant physiology.

Current genetic strategies such as the generation of transgenic and mutant plant lines in addition to genome-wide expression analyses of plants either under pathogenic or mutualistic interactions will help to unravel the regulatory networks that control PA metabolism in plants. The expression of genes of PA metabolism in transgenic lines should be driven by promoters specifically induced by microorganisms, since constitutive expression could offer undesirable characteristics. These studies will provide a better knowledge of the roles played by these biological amines in the development of plant–microbe interactions. It is conceivable that a deeper understanding in this field will make wonderful contributions to the design of better strategies to control plant diseases and favor beneficial plant–microbe associations.

## Conflict of Interest Statement

The authors declare that the research was conducted in the absence of any commercial or financial relationships that could be construed as a potential conflict of interest.
